# Laboratory Diagnosis of Animal Tuberculosis in Tracing Interspecies Transmission of *Mycobacterium bovis*

**DOI:** 10.3390/pathogens14050459

**Published:** 2025-05-07

**Authors:** Ewelina Szacawa, Nina Kozieł, Sylwia Brzezińska, Ewa Augustynowicz-Kopeć, Marcin Weiner, Krzysztof Szulowski, Monika Krajewska-Wędzina

**Affiliations:** 1 Department of Bacteriology and Bacterial Animal Diseases, National Veterinary Research Institute, Partyzantow 57, 24-100 Pulawy, Poland; nina.koziel@piwet.pulawy.pl (N.K.); kszjanow@piwet.pulawy.pl (K.S.); 2 Department of Microbiology, National Tuberculosis and Lung Diseases Research Institute (NTLD), Plocka 26, 01-138 Warsaw, Poland; s.brzezinska@igichp.edu.pl (S.B.); e.kopec@igichp.edu.pl (E.A.-K.); 3 Pope John Paul II State School of Higher Education, Sidorska 95/97, 21-500 Biala Podlaska, Poland; m.weiner@dyd.akademiabialska.pl; 4 Sub-Department of Microbiology, Department of Preclinical Veterinary Sciences, Faculty of Veterinary Medicine, University of Life Sciences, Akademicka 12, 20-033 Lublin, Poland; monika.krajewska@up.lublin.pl

**Keywords:** *Mycobacterium bovis*, MTBC, tuberculosis, animal tuberculosis, transmission, alpaca, cattle, horse, sheep

## Abstract

*Mycobacterium* is one of the most dangerous pathogens of both animals and humans. Bovine tuberculosis (BTB) is a disease caused by mycobacteria belonging to the *Mycobacterium tuberculosis* complex (MTBC), which spreads mainly among domestic cattle but also to mammals other than cattle. The transmission of MTBC between different species requires research and epidemiological investigations to control its spread. When multiple species are a reservoir of infection, it poses a significant public health and veterinary concern. In this study, the diagnosis of alpaca, cattle, horses, dogs, a sheep and a cat from one farm suspected of bovine tuberculosis was performed. The animals (except for one horse, the dogs and the cat) were euthanised after the intradermal tuberculin tests. Mycobacterial isolation from animal tissue samples was performed. The obtained *Mycobacterium* strains were genotyped using spoligotyping and mycobacterial interspersed repetitive unit–variable number tandem repeat (MIRU-VNTR) methods. The isolates from a horse, two cows, a sheep and an alpaca were classified as *Mycobacterium* (*M.*) *bovis*. The single *M. bovis* spoligotype SB0666 pattern was isolated, and the MIRU-VNTR results presented the same 222632237401435 patterns. The molecular investigation uncovered information on the relationship of *Mycobacterium bovis*.

## 1. Introduction

In 2009, Poland gained official bovine tuberculosis (BTB)-free status by the European Commission Decision 209/342/EC of 2009 and has maintained this status to date [1]. Despite the fact that the country is officially free of BTB, this zoonosis is considered to be endemic in the Bieszczady Mountains (Southern Poland, bordering Ukraine and Slovakia). Over the course of a quarter of a century, BTB has killed the “Górny San” herd of 45 bison in this region [2]. The last cases in wild boars were recorded in 2020–2023 [3]. From an epidemiological point of view, the transmission of MTBC between species is very important and requires research and epidemiological investigations to control it. In the case of BTB, the disease often affects almost all individuals in the herd [4]. Moreover, it is also possible to transmit the disease to mammals other than cattle, including domestic animals, wildlife and sylvatic species. Cases of the disease have been evidenced in, for example, badgers [5], wild boar [6], red deer [7] and alpacas [8]. In Central Europe, the above-mentioned species can pose a risk of BTB transmission by moving and feeding in various regions of the country, in cattle pastures and in forests near tourist areas. The transmission can be spread not only through close contact between infected and susceptible hosts and through aerosol droplets (during coughing or sneezing) but also in shared environments, i.e., through watering points and pastures. Interspecies transmissions might also occur on farms within domestic animal species. There are reports that *M. bovis* was isolated from goats, sheep, cats, dogs, pigs, farmed wild boars, llamas and alpacas. It is worth noticing that differences in species susceptibility exist; for example, cats seem to be more susceptible to infection than dogs, and, generally, acid-fast bacilli have been isolated very seldom from horses [9,10]. In fact, different farmed animals can be carriers of *M. bovis*, and there is a risk that the infection with MTBC may spread to humans [8,11,12]. Due to its zoonotic potential, it poses a significant public health and veterinary concern. BTB is a challenge to the TB control programme because it is difficult to eradicate when multiple species can be infected and act as reservoirs of infection on farms. A recent European Food Safety Authority (EFSA) publication from 2022 reported 130 cases of human tuberculosis due to *M. bovis* and *M. caprae*. Regarding cattle, the report indicates that seventeen EU Member States reached a disease-free status in terms of MTBC during 2022. Among the remaining states, there are disease-free status zones or provinces, including those in Italy, Portugal and Spain, and seven had no zones with disease-free status. According to the report, in 2022, in the EU, the overall proportion of cattle herds infected with MTBC was as low as 0.61%. Thirteen states detected no cases of bovine tuberculosis. The remaining countries reported bovine tuberculosis at various levels of prevalence at the national level. Infection with *M. bovis* and *M. caprae* was reported in Bulgaria, France, Germany, Ireland, Italy, Poland, Romania, the United Kingdom (Northern Ireland) and Austria. Countries with disease-free status zones also reported 149 cattle herds with MTBC infection, confirming that the detection of bovine tuberculosis in disease-free status zones exists [13].

From 2017 to 2019, we recorded an outbreak of tuberculosis in alpacas imported from Great Britain to Poland. The described transmission seems to be linked with a previous outbreak of BTB in alpacas in Poland in contact with “British” ones [8,14]. The objective of the study was to diagnose different animal species suspected of BTB and assess their epidemiological relationship. According to the “One Health” concept, animal tuberculosis should be especially monitored because such zoonosis can be transmitted to humans through milk product consumption, direct contact with carrier animals or the environment, causing a zoonosis.

## 2. Case Description

### 2.1. Materials and Methods

#### 2.1.1. Animals and Samples

Administrative proceedings at the farm located in Southern Poland were initiated in 2019 based on information provided by the Veterinary Inspectorate about the purchase of an alpaca from a herd infected with tuberculosis, delivered to the aforementioned farm in 2018 [14]. During the investigation conducted at the farm, it was established that two alpacas had died in 2018 and that the cause(s) of their deaths had not been identified. The owners of the animals did not know about the tuberculosis and therefore accepted the animals’ deaths as a natural state of affairs. No autopsies were performed on these individuals, and no organs were collected for further tests. In 2019, inspectors found one alpaca (offspring of an alpaca that had died in 2018), two cattle, two horses, two dogs, a sheep and a cat at this farm. The animals (except for one horse, the dogs and the cat) were euthanised after obtaining positive results of a single intradermal tuberculin test (SITT) or a comparative intradermal tuberculin test (CITT).

- Single Intradermal Tuberculin Test

The SITT is the standard method for the detection of tuberculosis in a wide variety of mammals and typically exhibits greater sensitivity than the CITT. It was carried out by intradermal injection of 0.2 mL bovine tuberculin (Bovitubal 28,000 IU/mL; Bioveta a.s., Ivanovice na Hané, Czech Republic) into a defined site. For the horses and cows, the examination was performed on the neck, and for the sheep, alpaca, dogs and cat, on the medial aspect of the left thigh. The test was read 72 h later by determining the increase in skinfold thickness in millimetres at the injection site according to the manufacturer’s instructions.

- Comparative Intradermal Tuberculin Test

The CITT measures the difference in increased skin thickness between bovine tuberculin purified protein derivative and that of avian tuberculin purified protein derivative, therefore providing greater specificity. In accordance with the instructions of the chief veterinary officer, after 42 days, the horse and cow were examined using the CITT according to the manufacturer’s instructions. The CITT was performed by intradermal injection of 0.2 mL bovine tuberculin (Bovitubal 28,000 IU/mL; Bioveta a.s., Ivanovice na Hané, Czech Republic) and avium tuberculin (Avitubal 28,000 IU/mL; Bioveta a.s., Ivanovice na Hané, Czech Republic) into a defined site on the right neck.

#### 2.1.2. Mycobacterial Isolation

Tissue samples from each animal were cut into small pieces and homogenised in a MiniMax tissue homogeniser (Interscience, Saint-Nom-la-Bretèche, France) for 3 min with the addition of 5% oxalic acid as a decontaminant. Next, the filtrate of the homogenate was incubated at 37 °C for 20 min to increase the decontamination efficiency, and after this incubation, it was centrifuged for 10 min at 3500× *g* (MPW 223e, MPW Med. Instruments, Poland). The pellet/sediment was washed twice with sterile physiological saline, and the material was plated on three Stonebrink and three Petragnani solid media (in-house). The culture was incubated at 37 ± 2 °C for six weeks and inspected for specific colonies once a week [8]. All the isolates had characteristic morphology and were positive for the Ziehl–Neelsen staining (Tb-color, Merck Millipore, Darmstadt, Germany). They were taken for subsequent identification and genotyping tests.

#### 2.1.3. Identification and Genotyping

- Spoligotyping

Spoligotyping is a PCR and hybridisation technique commonly used for genotype isolates of the *Mycobacterium tuberculosis* complex and measures polymorphism in the presence or absence of multiple short spacer units found in the Direct Repeat (DR) region of the chromosome. Spacers in DR region 43 were selected to be the target for the internationally standardised spoligotyping technique [15,16]. Genotyping based on spoligotyping (Ocimum Biosolutions, Hyderabad, India) was conducted according to the standardised protocol described by Kamerbeek [15] in a thermocycler (Veriti 96 Well Thermal Cycler, Applied Biosystems, Waltham, MA, USA) and a miniblotter (Mapmygenome Miniblotter 45, Interchim Innovations, Montluçon, France). The spoligotype patterns were compared to those of strains registered in the Spoldb4 database [17]. Spoligotypes were assigned according to international spoligotype nomenclature [16].

- Mycobacterial Interspersed Repetitive Unit–Variable Number Tandem Repeat

Mycobacterial interspersed repetitive unit–variable number tandem repeat (MIRU-VNTR) typing is widely used to evaluate the population structure, strain genetic diversity and transmission of the *Mycobacterium tuberculosis* complex [18]. The MIRU-VNTR was conducted manually for the 15 loci, according to Rodriguez [19], in a thermocycler (Veriti 96 Well Thermal Cycler, Applied Biosystems, Waltham, MA, USA). The following loci were analysed: MIRU-4, MIRU-10, MIRU-16, MIRU-26, MIRU-31, MIRU-40, VNTR 424, VNTR 577, VNTR 2165, VNTR 2401, VNTR 3690, VNTR 4165, VNTR 2163b, VNTR 1955 and VNTR 4052. A Taq DNA polymerase kit (EurX, Gdańsk, Poland) was used for the PCR, and a 2% agarose gel (Merck, Warsaw, Poland) was used for the electrophoresis. The H37Rv strain was used as a positive control for the test. The samples were clustered based on the typing with the 15 loci, and the allele copy numbers were entered into a Microsoft Excel spreadsheet for analysis.

### 2.2. Results

#### 2.2.1. Clinical Examination (Including TST), Necropsy

At ante-mortem examination, no animals showed clinical signs suggesting health problems, nor were there any changes post-mortem. Among the tested animals, the horses, cows, sheep and alpacas were considered TST reactors (i.e., skinfold thickness change > 4 mm). The exact skinfold thickness results on the day of reading are presented in Table 1. Finally, the CITT result in the cow was interpreted as positive, and the animal was put down. In the 14-year-old mare, the result was finally determined to be negative (Table 2).

#### 2.2.2. Bacteriology and Genetic Analysis

The isolates from a horse, two cows, a sheep and an alpaca were classified as *Mycobacterium bovis*. Hybridisation patterns obtained by spoligotyping were transcribed into octagonal codes, which were entered into international spoligotype databases. The single *M. bovis* spoligotype SB0666 pattern was isolated (Table 3). The MIRU-VNTR results of the five strains presented the same 222632237401435 pattern.

## 3. Discussion

Although the cross-species transmission of tuberculosis on a single farm is known in the veterinary community, this phenomenon is rarely documented. Most publications concern *M. bovis* isolated from goats, sheep, cats, dogs, pigs, farmed wild boars, llamas and alpacas. The majority of such cases have not been tested for the direct transmission of *M. bovis* to other species, mainly because the standard procedures of BTB testing do not include an investigation of the relationship of the isolates in farms [9,10]. In the UK, cases of BTB in alpacas were reported in recent years. It is the country from which most of the alpacas correlated with this study were imported to Poland, but limited data exist about spoligotypes obtained recently from alpacas in the UK [20]. There are also studies describing *M. bovis* infection within the same farm, but mostly, there are descriptions of transmission of the infection from cattle to other animal species, for example, the confirmed cases of tuberculosis in a sheep flock that was kept alongside a cattle herd infected with *M. bovis* in Ethiopia [21]. Most of the publications concern the hypothetical relationship between the *M. bovis* isolates based on genotyping of isolates derived from different localisations, as described by Barandiaran et al. regarding *M. bovis* infection in pigs slaughtered in Argentina and typed with spoligotyping. As a result, it occurred that pigs shared some of the spoligotypes with cattle [10]. The BTB case described in our study reported the transmission of *M. bovis* from alpacas to three other animal species on the same farm, i.e., a horse, a cow and a sheep. This was proven by two different genotyping methods. Moreover, the same MIRU-VNTR and spoligotype patterns were obtained in other alpacas from herds in Poland that were related to those from our study. In the past, some of the alpacas from this herd were purchased and introduced into the examined herd [8]. According to the international database of bovine spoligotypes, the pattern found in our study—SB0666—comes from Great Britain and was registered in 2003. The infection can be spread through shared pastures or housing animals together with infected animals. In a case report, an outbreak of *M. bovis* infection in a Lleyn sheep flock in the UK was reported, and no direct contact with cattle was noted, although BTB is endemic in this area. The transmission of the disease by the environment or by purchasing carrier animals is the most likely [22]. In another study, a steer and a sheep were infected with BTB after sharing the same air space. The same *M. bovis* genotype was revealed, and it proved the transmission from sheep to steer [23]. In Spain, cattle or goats appeared to be the source of BTB infection for sheep, as animals shared the facilities. The molecular analysis revealed the same pattern of isolated *M. bovis* strains [24]. Recently, there have been studies on an environmental transmission model lasting 4 years that incorporates a territory experiment and between-species transmission concerning badgers and cattle. It revealed that the environment can play a significant role in the transmission of BTB, and the half-life of *M. bovis* bacilli in the environment is around 177 days [25]. In the case described in our study, the infection could also have spread through contaminated feed, water or grass via alpaca excreting bacteria on shared grazing fields with the horses, cows and sheep. The spreading of the disease can also be facilitated by alpacas’ spitting behaviour. Although cattle are the main host of *M. bovis*, animal species such as horses and sheep are also susceptible to these bacteria [26]. Although horses are thought to be more resistant to MTBC infections when compared to other livestock animals, and the incidence of tuberculosis in this species is extremely low, it is possible for horses to be infected as well, especially when such animals are living in close contact with a BTB-infected animal [27,28]. The incidences of infections in horses were summarised in a review by Pavlik [29]. It is worth mentioning that the presence of tuberculosis caused by genotypically identical strains of *M. bovis* in sheep and horses was described for the first time in Poland.

Because bovine tuberculosis has the potential for transmission to humans, especially those with various health disorders, in this case, investigations were also performed on the exposed people. Alpacas are a very popular species used on farms in children’s therapy. The owner of the farm bought alpacas for this very purpose because she had a grandson who had leukaemia. Close contact with the animals was supposed to help him recover. Fortunately, the boy did not become infected. The child underwent a tuberculin test and an IGRA test [30], both of which were negative. The boy was under the care of specialist clinics the entire time.

The transmission of *M. bovis* to animal species other than cattle complicates the control measures in cattle. Systematic research based on different techniques is needed to effectively control the disease. In the epidemiology of diseases caused by acid-fast mycobacteria, rapid detection and identification of the pathogen are important. The intradermal screening test is the first stage of the research. Next, a post-mortem examination and culture of pathogenic bacteria are useful methods to assess disease surveillance. Screening animals using the tuberculin test provides information about the infection in an animal within 72 h. This is beneficial because, based on this result, you can separate animals suspected of having the disease from healthy animals. Undoubtedly, microbiological methods are the gold standard in diagnosing tuberculosis. When tuberculosis is suspected, a bacteriological examination should be performed. The result of a bacilli growth can be obtained after about 6 weeks [9,31]. The identification of strains can be performed with species-specific PCR targeting, i.e., insertion sequences such as IS6110 or IS1081 [32]. Based on the strains determined, molecular identification can be performed using typing methods such as VNTR and spoligotyping; often, both methods are used to increase the discriminatory power of MTBC genotyping [15,33]. Direct PCR on tissue samples is thought to be a good alternative to the bacteriological culture. It is a good approach to obtain fast and reliable detection of MTBC species when distinguishing between individual species within this complex is not necessary [34]. Recently, whole genome sequencing has become a valuable alternative in epidemiological analyses [35]. It provides information on the origin of the strain, its relationship and molecular diversity. The epidemiological investigation provides information on the possible relationship between mycobacteria found in different animals. Using molecular examinations of the environment, soil, feed and potential infection sites can be indicated [36,37]. Identifying such risk factors will make it possible in the future to limit, to some extent, the spread of tuberculosis among animals and even the transfer of infection from animals to humans.

## 4. Conclusions

Epidemiological investigations into BTB in different species are essential to control its spread. In this study, all isolates derived from a horse, two cows, a sheep and an alpaca were classified as *M. bovis*. The single *M. bovis* spoligotype pattern was isolated, and the same MIRU-VNTR pattern was obtained. Therefore, the molecular investigation provided information on the relationship of the analysed mycobacteria with previous outbreaks of BTB in alpacas.

## Figures and Tables

**Table 1:** Results of individual animals in SITT.

Animal Species, Age, Sex	Skin Fold Thickness Before Tuberculin Supervision (In mm)	Skin Fold Thickness After 72 h (In mm)	Nature of Reaction/Result
mare, 14 years old	4.0	5.0	limited hard/negative
mare, 8 years old	4.13	8.0	diffuse painful infiltration/positive
cow, 2 years old	6.6	13.0	limited hard/positive
cow, 6 months old	6.0	6.8	limited hard/negative
sheep (female), 6 years old	no measurement	no measurement	limited hard/positive
alpaca (female), 6 years old	no measurement	no measurement	diffuse infiltration, diameter 5 cm/positive
dog (female), 5 years old	no measurement	no measurement	no reaction/negative
dog, (female), 8 years old	no measurement	no measurement	no reaction/negative
cat (female), 6 years old	no measurement	no measurement	no reaction/negative

**Table 2:** Results of individual animals in CITT.

Animal Species, Age, Sex	Type of Tuberculin	Skin Fold Thickness After 72 h (In mm)	Skin Fold Thickness After 72 h (In mm)	Nature of Reaction/Result
mare, 14 years old	Bovitubal	3.0	3.1	no reaction/negative
	Avitubal	3.5	4.0	no reaction/negative
cow, 6 months old	Bovitubal	9.5	12.5	limited hard/questionable
	Avitubal	10.0	10.5	no reaction/negative

**Table 3:** Typing of strains using spoligotyping.

Animal Species	Hybridisation Pattern of the Strain	Octagonal Code	Spoligotype (Mbovis.org Database)	Spoligotype (SITVIT Database)
horse	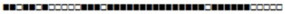	664073777757600	SB0666	*M. bovis* _2 678
cow 1	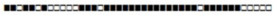	664073777757600	SB0666	*M. bovis* _2 678
cow 2	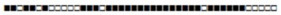	664073777757600	SB0666	*M. bovis* _2 678
sheep	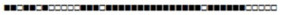	664073777757600	SB0666	*M. bovis* _2 678
alpaca	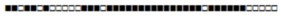	664073777757600	SB0666	*M. bovis* _2 678

## Data Availability

All the data supporting our findings are contained within the manuscript. The raw datasets analysed during the current study are available from the corresponding author on reasonable request.

## Abbreviations

The following abbreviations are used in this manuscript:

BTB	Bovine tuberculosis
*M. bovis*	*Mycobacterium bovis*
EFSA	European Food Safety Authority
MTBC	*Mycobacterium tuberculosis* complex
SITT	Single intradermal tuberculin test
CITT	Comparative intradermal tuberculin test
MIRU-VNTR	Mycobacterial Interspersed Repetitive Unit–Variable Number Tandem Repeat
DR	Direct Repeat
